# The Effect of Direct Electric Current on the Plastic Behavior of AA7075 Aluminum Alloy in Different States of Hardening

**DOI:** 10.3390/ma14010073

**Published:** 2020-12-25

**Authors:** Daniel Dobras, Stefania Bruschi, Enrico Simonetto, Małgorzata Rutkowska-Gorczyca, Andrea Ghiotti

**Affiliations:** 1 Department of Metal Forming and Metrology, Wrocław University of Science and Technology, 7-9 Ignacego Łukasiewicza Street, 50-371 Wrocław, Poland; 2 Department of Industrial Engineering, University of Padova, Via Venezia 1, 35131 Padua, Italy; stefania.bruschi@unipd.it (S.B.); enrico.simonetto.1@unipd.it (E.S.); andrea.ghiotti@unipd.it (A.G.); 3 Department of Automotive Engineering, Wrocław University of Science and Technology, Wyb. Wyspiańskiego 27, 50-370 Wrocław, Poland; malgorzata.rutkowska-gorczyca@pwr.edu.pl

**Keywords:** aluminum alloy, formability, electrically-assisted forming, electroplastic effect, electron wind theory, Portevin–Le Chatelier effect

## Abstract

Electrically-Assisted Forming (EAF) techniques are interesting and promising for the automotive industry. Electrically-assisted tensile tests were carried out on specimens of AA7075 aluminum alloy in different states of hardening, namely T6 (the as-received state) and the supersaturated solid solution state. All the tests were carried out in quasi-static conditions under the application of direct electric current (DC) in the range of 90 to 540 A. The experimental results showed that with a DC density of 10 A/mm^2^ the uniform strain and strain at fracture increased when the AA7075 was in the supersaturated solid solution state. A correlation between the mechanical results and microstructural features analysed through transmission electron microscopy was assessed. An explanation of the investigated phenomena based on the electron wind theory, heterogeneous microscale Joule heating and the Portevin–Le Chatelier (PLC) effect was finally proposed.

## 1. Introduction

In recent years, high-strength aluminum alloys have become very popular in the automotive industry thanks to the significant advantages they offer, such as high specific strength, good weldability, corrosion resistance, and relatively good formability [1,2]. The use of aluminum alloy sheets for the car’s body structure can be enough to greatly reduce the weight and therefore the fuel consumption [3], especially in the case of high-strength aluminum alloys such as the 7000 series. However, their formability at room temperature is relatively low, which forces manufacturers to carry out the stamping processes under warm or even hot forming conditions. Warm forming is applied when the alloy is in the T6 temper state in order to maintain the high-strength properties after the process, but the increase in formability under such a temperature range can be insufficient for manufacturing complex-shaped parts. On the other hand, hot forming is usually carried out after the alloy solubilization treatment, thus allowing a drastic formability increase, but this procedure imposes a final artificial aging to improve the part’s strength. In general, warm and hot methods have other drawbacks, such as higher energy consumption, and increased die wear due to the more significant adhesion phenomena between the die and the sheet [4].

In this context, the exploitation of Electrically-Assisted Forming (EAF) techniques can help with avoiding the above-mentioned shortcomings. Several studies have shown that the material flow stress can be reduced and its ductility increased when an electric current is applied to the metal during plastic deformation. Conrad [5] discovered that in some pure metals, such as aluminum, copper and zinc, the flow stress could be reduced by the application of short electric current pulses, but the current density had to be higher than a threshold value. He claimed that the flowing current generated an electron wind, which affected the dislocation motion, causing their acceleration. This effect was named the electroplastic effect (EPE). Roh et al. [6] investigated the behavior of the AA5052 aluminum alloy under a pulsed electric current, showing a significant increase in the fracture strain in the samples under tension. Kim et al. [7] showed that, in the case of the same aluminum alloy, the specimens which were pre-strained showed higher elongation than the ones without the effect of pre-straining during EA(Electrically-Assisted) tension. Similar results were obtained for other alloys of the 5000 series [8,9]. Kim et al. [10] reported that, during uniaxial tension tests, electrically-assisted annealing of the aluminum alloy could occur, which could be responsible for a better plasticity of the material. Ross et al. [11] reported that the flow stress could be significantly reduced by applying direct current during deformation; the formability of the tested specimens was, however, quite low due to its very high temperature and its heterogeneous distribution and hence the heterogeneous deformation of the specimens. However, Breda et al. [12,13], through conducting tensile tests with direct current on the AA1050 pure aluminum, proved that the strain at fracture could be increased. Ruszkiewicz et al. [14,15] studied the influence of direct and pulsed current on the mechanical properties of the AA7075-T6 aluminum alloy. They proposed a new electron stagnation theory to describe the behavior of the alloy during deformation with a simultaneous application of electric current. Other studies focused on various plastic deformation processes, such as compressing [16] or wire drawing [17], showing several advantages when electrically-assisted.

In the present study, the effect of Direct Current (DC) on the plastic behavior of the AA7075 aluminum alloy in different states of hardening was investigated and assessed in relation to Transmission Electron Microscopy-observed microstructural features. Contrary to other studies [14,16], where usually materials in the hardening state were tested, this paper also focuses on the supersaturated solid solution state. Furthermore, an explanation of the observed phenomena based on the electron wind theory, heterogeneous microscale Joule heating and Portevin–Le Chatelier (PLC) effect was proposed.

## 2. Materials and Methods

AA7075-T6 aluminum alloy sheets with 1.5 mm thickness were used for the tests. The chemical composition of the as-received material, measured through a chemical composition analyser emission spectrometer LECO™ GDS500A (LECO Corporation, Saint Joseph, MI, USA), is given in Table 1. Table 2 shows the material Young modulus (E), yield strength measured for a 0.2% strain (Y_0.2_), ultimate tensile strength (UTS) and percentage elongation at break (A) in the as-delivered condition.

**Figure 1:**
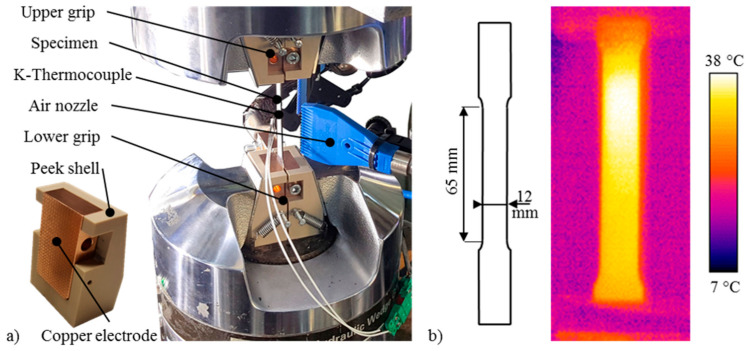
(**a**) Experimental set-up for the tensile tests and (**b**) sample geometry and example of temperature distribution in the sample (AA7075-SS 5 A/mm^2^).

The testing samples in a dog-bone shape (see Figure 1b) were milled from the as-received sheets, parallel to the rolling direction, with 65 (±0.2) mm nominal gage length, 12 (±0.1) mm nominal gage width and an initial cross-section area (A_0_) of 18 (±0.2) mm^2^, following the ISO 6892 standard. In order to obtain a different state of hardening, a heat treatment was conducted: heating at 480 °C for 30 min in an oven and immediate water quenching were applied in order to obtain the Super Saturated (SS) state of the AA7075 aluminum alloy.

Uniaxial tensile tests were conducted using a 50 kN MTS™ hydraulic dynamometer (MTS Systems Corporation, Eden Prairie, MN, USA), not later than 20 min after supersaturation. During testing, DC was applied to the sample using specially designed insulated grips, with copper electrodes embedded in a polyether ether ketone (PEEK) shell, as shown in Figure 1a. A DC generator with a maximum power of 60 kW, maximum voltage of 10 V and maximum current of 6 kA was used to provide the electric power. The applied current was in the range from 90 to 540 A. Quasi-static tensile tests were performed with a constant strain rate of 0.1 s^−1^ until fracture. For each parameter set, three samples were tested to verify the repeatability of the results. Table 3 shows the experimental plan and the testing parameters.

The sample temperature was monitored by a FLIR™ A40 infrared thermal imaging camera (FLIR Systems, Wilsonville, OR, USA), previously calibrated by taking as reference the temperatures measured by a k-type thermocouple spot welded on the sample. The sample was coated with a black and white dot stochastic pattern in order to record its deformation using a high-speed CCD camera (Allied Vision-Pike, Stadtroda, Germany). The pictures taken by the CCD camera were processed using the GOM™ Aramis software (V8, GOM, Braunschweig, Germany) and then the strain values, namely the strain at fracture and uniform strain, were calculated at the gauge length. Multiple air nozzles were set up as shown in Figure 1a and compressed air at approximately 10 bars was used to cool the samples during the tests in order to reduce influence of the Joule heating, and hence to validate the so-called pure EPE.

A laboratory microscope (LEICA Microsystems, Wetzlar, Germany) was used to measure the area of the fracture (Af) of the tested samples in order to have a further indication of the strain at fracture reached by the samples. The microstructural observations of the specimens from the tensile test were performed using Transmission Electron Microscopy (TEM). Thin foils were prepared through mechanical polishing to around 70 µm of thickness; then, using a mechanical guillotine, 3 mm diameter discs were cut out. The discs were electrolytically polished and then ion polished using a GATAN™ DuoMill device (Corporate Headquarters, Pleasanton, CA, USA). The bright-field images were obtained with a Hitachi™ H800 TEM (Hitachi, Tokyo, Japan) operating at 150 kV. The fracture surfaces were observed with a Phenom™ X SEM (Thermo Fisher Scientifi, Waltham, MA, USA) operating at 15 kV.

## 3. Results

### 3.1. Thermo-Electrical Results

Table 4 shows the average values of the maximum temperatures reached by the samples during the tensile tests as a function of the applied DC density (defined as the DC intensity divided by the nominal cross section area of the sample in the gauge length). The highest temperature of 121 (±2) °C was measured when using a DC density of 30 A/mm^2^ on the AA7075 sample in the T6 state. However, this temperature is still much lower than the ones of the usual warm forming range for AA7075 (200–250 °C). Figure 1b shows that the temperature distribution was nonuniform during the test along the sample gage length. Note that this is a common effect in electrically-assisted processes [9], as the copper grips tend to cool down the specimen under the gripping zone as their temperature is lower due to their higher mass and electric conductivity.

### 3.2. Mechanical Results

**Figure 2:**
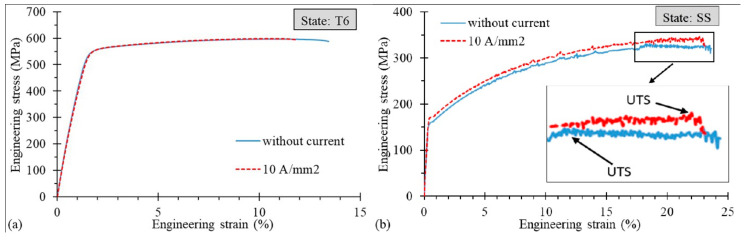
Engineering stress–strain curves for the AA7075-T6 (**a**) and SS (**b**) states.

Examples of engineering stress–strain curves with the application of DC for the T6 and SS state are shown in Figure 2a,b, respectively. The experimental results in Figure 2b show the influence of the 10 A/mm^2^ application on the uniform strain (*ε_UTS_)*, which is clearly higher than in the case without the current, even if the fracture strain (*ε_f_*) is reached at the same level. Therefore, the application of 10 A/mm^2^ increases the amount of strain required for the necking occurrence without a significant increase in the required strain at fracture. However, different results are presented in Figure 2a, where the values of both *ε_f_* and *ε_UTS_* are slightly lower than in the case without current application. Note that the Portevin–Le Chatelier (PLC) effect, which manifests itself in the form of drops and serrations on the stress–strain curves, occurred in the case of the SS state.

**Figure 3:**
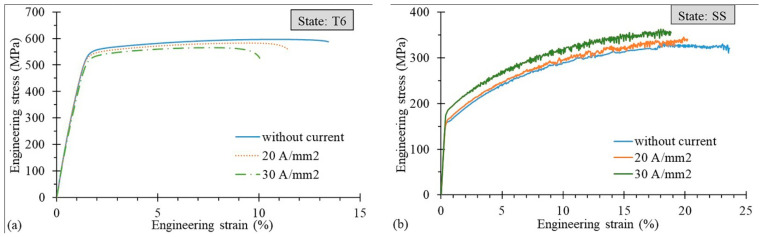
Engineering stress–strain curves for the AA7075-T6 (**a**) and SS (**b**) states at varying current density applications.

Figure 3a shows the engineering stress–strain curves of the T6 state with and without current application: the current application caused not only a decrease in the elongation, but also a UTS decrease. On the contrary, in the case of an SS state, the DC application determined the UTS increase and the extent of the amplitude of the serrations and its range of occurrence (Figure 3b), but, at the same time, a decrease in the strain at fracture was noted.

**Figure 4:**
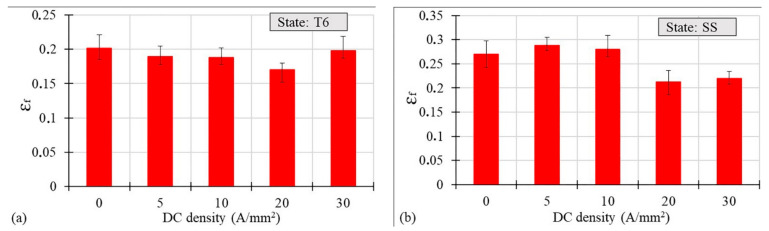
True strain at fracture vs. DC density for the AA7075-T6 (**a**) and SS (**b**) states.

**Figure 5:**
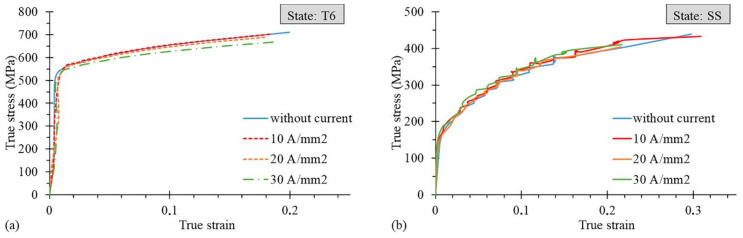
True stress–true strain curves for the AA7075-T6 (**a**) and SS (**b**) states at varying current density application.

Figure 4a,b plot the true strain at fracture *ε_f_* values as a function of the current density for the T6 and SS states, respectively, measured using the Aramis system. In the case of the T6 state, the application of DC determined a decrease in *ε_f_* till the value of 20 A/mm^2^, which represented the worst condition for the elongation at rupture, while it rose for 30 A/mm^2^, where *ε_f_* reached the same value of the testing condition without DC; whereas in the latter conditions the samples reached the highest temperatures seen during the test. On the contrary, in the SS state, for 5 and 10 A/mm^2^, a very slight increase in *ε_f_* was observed compared to the testing conditions without current application, followed by a rapid significant decrease below the reference value. Figure 5a,b show an example of the true stress-true strain curves of the T6 and SS states obtained with and without current application.

**Figure 6:**
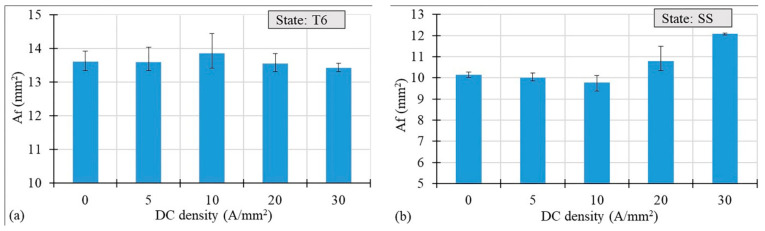
Area at fracture vs. DC density for the AA7075-T6 (**a**) and SS (**b**) states.

Figure 6a,b show the area at fracture *A_f_* values as a function of the current density. There are small differences at varying current density in the case of the T6 state. However, in the case of the SS state, for current densities higher than 10 A/mm^2^, the area at fracture increased significantly (Figure 6b), which suggests a decrease in the material’s maximum deformation at fracture ($\varepsilon_{f}=\ln\;\left(A_{0}/A_{f}\right)$).

**Figure 7:**
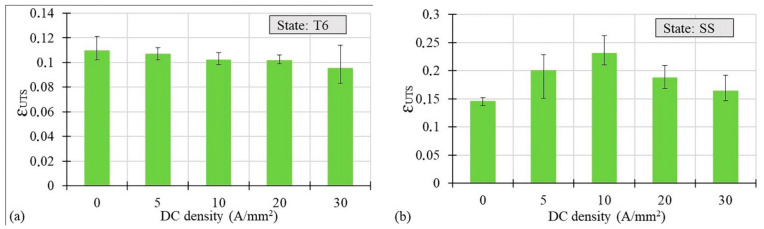
Uniform strain vs. DC density for the AA7075-T6 (**a**) and SS (**b**) states.

Figure 7a,b demonstrate the effect of the applied current density on the material’s uniform elongation in terms of *ε_UTS_*, namely the strain at Ultimate Tensile Strength (UTS). In the case of the T6 state, at increasing current density, a slight decrease in the uniform strain was observed. However, in the case of the SS state, a substantial increase in the value of *ε_UTS_* up to 10 A/mm^2^ was observed in comparison to the testing condition without current application. In this case, a decrease in the uniform strain occurred, but still it is at a higher point than for the reference. It is worth underlining that such changes of *ε_UTS_*, in the case of the SS state, cannot be explained by the very limited temperature increase reported in Table 4.

**Figure 8:**
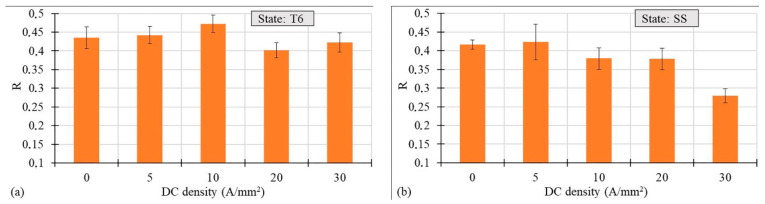
R-value vs. DC density for the AA7075-T6 (**a**) and SS (**b**) states.

Figure 8a,b present the DC density influence on the Lankford coefficient (R-value) calculated according to equation 1, for both the T6 and SS states, respectively:

(1) $$R=\;\frac{\varepsilon_{w}}{\varepsilon_{t}}$$

where $\varepsilon_{w}$ and $\varepsilon_{t}$ are the strains along the width and thickness direction respectively. In the case of the T6 state a small increase was observed till the value of 10 A/mm^2^, then the decrease in the R-value was observed. However, in the case of the SS state, the gradual decrease in the Lankford coefficient was observed at increasing current density, with a sharp drop for a DC density of 30 A/mm^2^.

**Figure 9:**
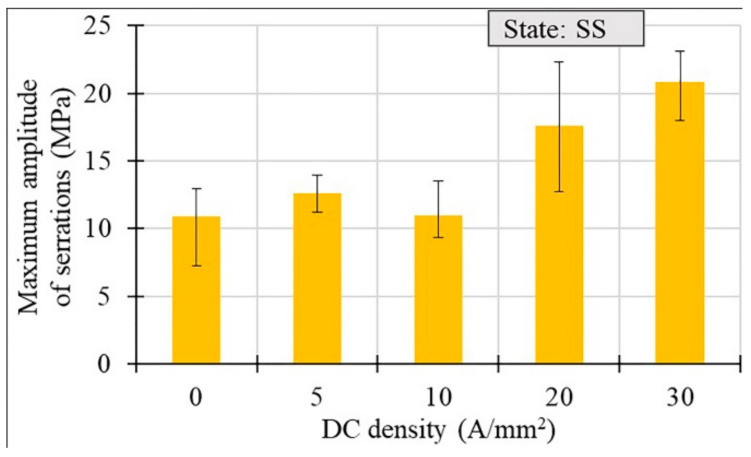
Maximum amplitude of serrations vs. DC density for the AA7075-SS.

The influence of the applied current density on the maximum amplitude of the serrations (defined as a maximum single stress drop taken from the engineering stress–strain curves), in the case of the SS state, is shown in Figure 9. It is clearly seen that the values of the maximum amplitude increased significantly when the applied current densities were at least 20 A/mm^2^. Over this threshold value the material also exhibited a fall in the true strain at fracture (Figure 4b) and consequently an increase in the area of fracture (Figure 6b).

### 3.3. Microstructural Results

**Figure 10:**
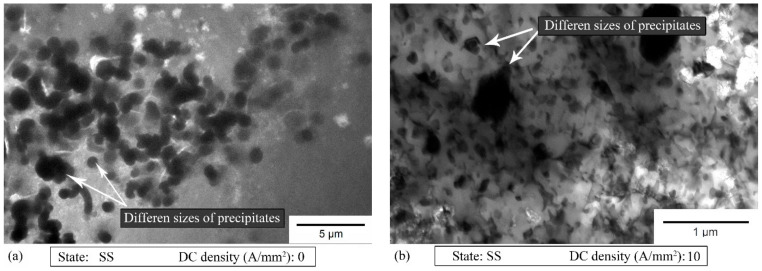
TEM bright-field images of the specimen strained to its maximum uniform strain without current (**a**) and with 10 A/mm^2^ density current application (**b**).

**Figure 11:**
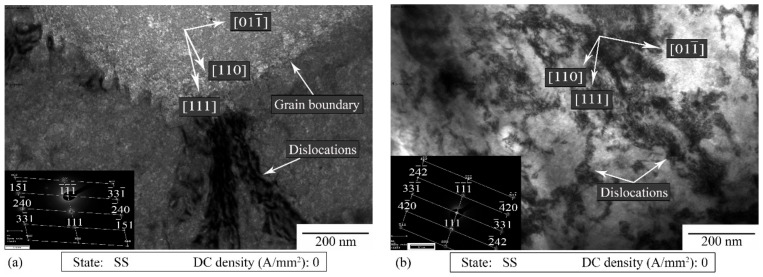
TEM bright-field image of the specimen strained without current to its maximum uniform strain (**a**) and to fracture (**b**).

In order to explain the increase in the uniform strain and the differences in the results for the SS state, TEM investigations were carried out. Four samples in the SS state were allocated for the TEM analysis, two strained to the value of the maximum uniform strain and two strained to fracture. From the first group, one was strained without current application to its maximum uniform strain of 0.145 (Figure 10a) and the other with 10 A/mm^2^ density current application to its maximum uniform strain of 0.23 (Figure 10b). From the second group, one sample was strained to fracture without current application (Figure 11a,b) and the other to fracture with 10 A/mm^2^ density current application.

In each analysed specimen the presence of coagulated precipitates in form of a cluster was observed. Additionally, both the arrangement of the precipitates inside the clusters and the arrangement of the clusters in the matrix were found to be uneven and chaotic. The size of most precipitates was in the range up to 2 μm. However, precipitates smaller than 200 nm were also observed (Figure 10b). Note that the TEM analyses were carried out more than one month after supersaturation and hence the natural ageing process affected the microstructural results, from the viewpoint of the precipitates. Nevertheless, the dislocation density analysis brought valuable information about the material behavior.

Figure 11a,b show the TEM images of the specimens strained without current application. Several defects in the form of single dislocations and a chaotic forest of dislocations were observed. The TEM images suggest that the dislocation density was higher in the case of the specimens strained without current application. Additionally, dislocations were observed in many forms, such as a pile up of dislocations in front of the grain boundaries and tangled dislocations around the precipitates as well as linear and longitudinal groups of dislocations.

Based on the analysis of the diffraction patterns and interpretation of stereographic projections, the zone axis of the diffraction patterns and orientations of thin film planes were determined. In the registered diffraction areas that are visible in Figure 11a,b, the orientations of the dislocation slip directions were determined based on their stereographic projections ([110] in Figure 11a and [110] in Figure 11b). The determined crystallographic directions confirmed the dislocation directions in the tested samples.

**Figure 12:**
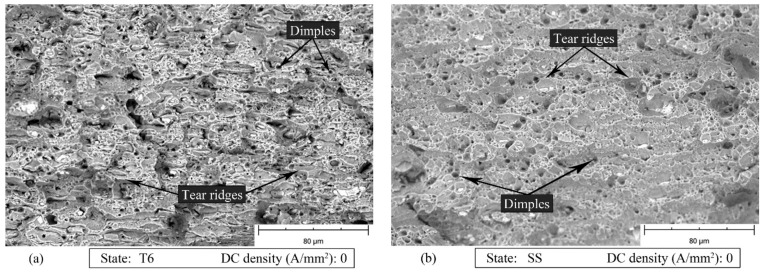
SEM fracture surfaces of the specimen strained without current application for the AA7075-T6 (**a**) and SS (**b**) states.

**Figure 13:**
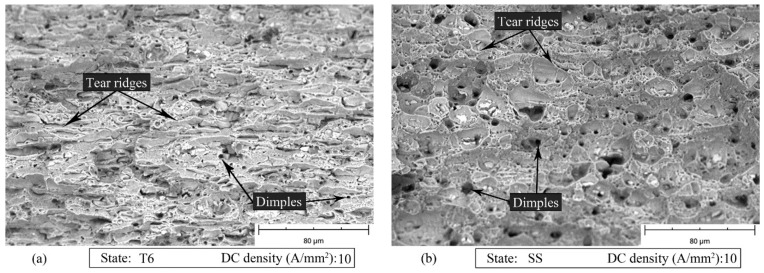
SEM fracture surfaces of the specimen strained with 10 A/mm^2^ current density application for the AA7075-T6 (**a**) and SS (**b**) states.

**Figure 14:**
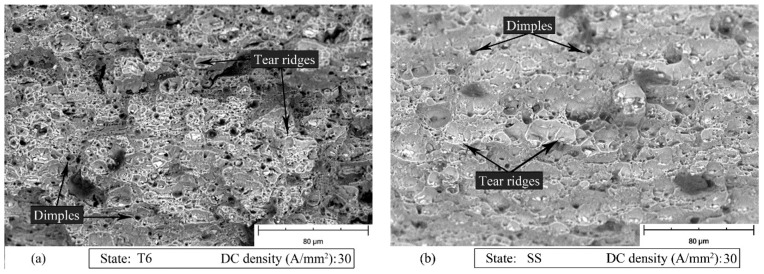
SEM fracture surfaces of specimen strained with 30 A/mm^2^ current density application for the AA7075-T6 (**a**) and SS (**b**) states.

Figure 12, Figure 13 and Figure 14 show the SEM fracture surfaces of the specimens tested without current, with 10 and 30 A/mm^2^ current density for both the T6 and SS states, respectively. In the case of the T6 state, the applied current did not significantly influence the fracture surfaces. The specimen strained with 10 A/mm^2^ current density application (Figure 13a), in comparison to the one strained without current application (Figure 12a), had only a few longer and flatter tear ridges. However, when 30 A/mm^2^ was applied (Figure 14a), the tear ridges were more oval and the number of dimples increased. The above-mentioned differences could be caused by the influence of the temperature, which globally reached the value of 121 °C.

In the other case, when the current is applied to the material in the SS state, 10 A/mm^2^ were sufficient to increase the dimple size and obtain longer and more oval tear ridges as shown in Figure 13b compared to Figure 12b. It is worth mentioning that in the SS state the maximum temperature reached with a current density of 10 A/mm^2^ was only 22 °C higher than room temperature. The dimple size decreased when 30 A/mm^2^ was applied. However, the morphology of the tear ridges remained unchanged (Figure 14b).

## 4. Discussion

### 4.1. Electron Wind Theory

It is commonly known that electrons can interact with dislocations and hence affect the movement of dislocations (dislocation line), thus the electron wind created by the flowing electric current gives an additional extra force, namely the electron wind force, which helps the dislocations to overcome obstacles [5]. Considering the two hardening states of the AA7075 samples that were the object of this study, dislocations have to overcome many more obstacles in the T6 states characterized by the presence of a large number of precipitates than in the SS state. Nevertheless, the applied DC densities in the T6 state were too limited to help the dislocations overcome the obstacles, namely the electron wind force acting on the dislocation line was too low. This is the reason why, regardless of the amount of the applied current density, both the strain at fracture and uniform strain were lower compared to the specimens tested without the current application (see Figure 4 and Figure 7). Even if it caused a significant temperature increase, any increase in the current density did not improve the material ductility, which instead led to the premature failure of samples [8,11,18]. On the other hand, the temperature increase can be considered responsible for the UTS decrease, as shown in Figure 10a. The flowing current in the material causes the electron–precipitate interaction and, together with the temperature increase, causes dissolution of the precipitations and hence the decrease in the UTS. It is worth mentioning that, according to Conrad [5], there is a threshold value of the current density below which the electroplastic effect does not occur. This threshold value for the aluminum alloys was set around 200 A/mm^2^, but this was found in the case of pulsed electric current, whereas in the case of direct current application no studies are available reporting any threshold value. However, in [13] it was shown that material ductility could be improved by applying DC current for values of current density lower than the above-mentioned ones. Nevertheless, after the precipitation hardening process, the materials seem insensitive to the application of electricity during their plastic deformation from the viewpoint of an increase in their ductility.

### 4.2. Heterogeneous Microscale Joule Heating

Kim et al. [7] proved that the pre-strain of samples made of 5xxx series aluminum alloys could increase the strain at fracture during the electro-pulsed tensile tests. They also showed an increase in the temperature with an increase in the pre-strain value (increase in the dislocation density). The current flowing through the material affects not only the crystal lattice, but also the defects of the crystal lattice, especially the pile up of the dislocations, grain boundaries, microcracks and voids. These interactions of the current with the lattice imperfections lead to a microscale increase in the temperature, the so-called localized Joule heating, because they are characterized by a higher electrical resistivity [14]. This type of interaction improves the dislocation motion and thus the material ductility. Taking into account the results of the tensile tests carried out on the alloy in the SS state, it can be said that the above-mentioned interactions occurred. In the case of the SS state, the pile up of dislocations increased, leading to a more and more intensive interaction between the electrons and the dislocations. Indeed, the heterogeneous microscale Joule heating occurred, leading to an improved dislocation motion and annihilation, and hence an improved material ductility (both *ε_f_* and *ε_UTS_* increased) as well as decreased dislocation density. The microstructural analysis proved that the dislocation density was the highest in the case of the SS state strained without the current (Figure 11a,b). Additionally, as shown in Figure 4b, the application of DC densities higher than 10 A/mm^2^ led to a premature failure of the samples and hence to a decreased strain at fracture as well as uniform strain. It is commonly known that during the electrically-assisted tensile test nonuniform distribution of the temperature occurs along the sample gage length (Figure 1b). Moreover, when the energy (in the form of the electrical energy) transferred to the specimen is so high, it can lead to a situation when the deformation zone is local (usually in the middle of the sample) and cannot be transferred to the other parts of the strained sample. As for example reported in [6,11,18], the total elongation of the material decreased, even if an increase in the temperature caused an increase in the material ductility. Taking into account the electron–void interaction, it is commonly known that the plastic deformation generates a large number of dimples and voids. However, the current flowing through the sample inhibits the growth of voids and dimples and their nucleation, because of the thermal stresses [19]. These effects are more significant when the current density is higher. Finally, the above-mentioned interaction leads to an improvement of material plasticity (e.g., the total elongation). On the other hand, the presence of bigger dimples and longer tear ridges was observed after 10 A/mm^2^ current density application (Figure 13b). As reported in [18] the above-mentioned morphology of the fracture surface testifies to the occurrence of ductile fractures. Taking into account that in this case the temperature only increased by about 22 °C, the observed phenomenon could been caused by the action of the flowing current.

### 4.3. Portevin–Le Chatelier Effect

It is commonly known that the PLC effect causes a decrease in the metal ductility [20]. The PLC effect manifests itself as serrations (jerky flow) on the stress–strain curves of many alloys and metals, such as copper, aluminum and steel. The most popular explanation of the PLC effect is based on the Dynamic Strain Aging (DSA) phenomenon, which is the result of the interaction between the moving dislocations and diffusing solute atoms (Cottrell atmosphere). However, many studies have shown that the PLC effect can be explained not only by the interaction of the mobile dislocations with the dissolved alloying atoms, but also with the vacancies, obstacles (different than solute atoms) or dislocation self-interaction [20,21]. As shown in Figure 3b and Figure 9, an increase in the applied current density in the SS state caused an increase in the UTS, the amplitude of the stress drop in the stress–strain curves and the range of the occurrence of the PLC effect (serrations) on the stress–strain curves (the onset strain of serrations). Taking into account the fact that the PLC effect caused a decrease in the ductility and that its effects were more significant with the increase in the applied current density (Figure 3b), it can be said that it is in contradiction to the electroplastic effect, which, despite the above-mentioned facts, caused an increase in the strain at fracture and uniform strain. However, the effect of the flowing current is stronger than the PLC effect up to a density of 10 A/mm^2^ DC. Note that in the T6 state (Figure 3a) an increase in the applied current density caused an inverse effect, namely a decrease in the UTS. This means that in the SS state a current-induced hardening effect was observed [22,23]. However, the previous studies of the PLC effect in the Al-Mg alloys [9,22,24] showed that an increase in the current density caused a decrease in the range of the occurrence of serrations and, finally, a complete suppression of the jerky flow on the stress–strain curves. Thus, it must be another, additional mechanism responsible for this difference in the aluminum alloys of the 7xxx series in the SS state. Based on the above-mentioned studies [9,22,24], the following is known: on one hand, the application of direct current and increase in the current density accelerate the mobility of the diffusion of the solute atoms and reduce their chance of interaction with the dislocations; on the other hand, the flowing current affects the small precipitates, causing their dissolution and hence a chance for their interaction with the mobile dislocations. However, in the tested samples in the SS state, there were no precipitates at the beginning of the tests, because they were conducted immediately after supersaturation. Moreover, the temperatures occurring during the tests (Table 4) promoted the aging process [25,26]. Thus the flowing current did not result in the dissolution of the precipitates, but promoted their growth (and nucleation) and hence increased the UTS and the range of the occurrence of serrations as they interacted with the dislocations [27,28]. A confirmation of the above-mentioned considerations is represented by the results from the TEM images (Figure 10). The microstructural analysis proved that many clusters of precipitates occurred, which, in their early stages, could be responsible for the jerky flow during the tests. However, researchers have claimed that the precipitates in the early stages have little effect on the serrations [21,27,28], so further studies, in a wider range of current densities and thus maximum reached temperatures, will be conducted.

## 5. Conclusions

The effect of direct electric current on the mechanical properties and plasticity of the AA7075 aluminum alloy in different states of hardening was experimentally investigated. It was shown that the application of 10 A/mm^2^ DC density could significantly increase the uniform strain in the supersaturated solid solution state. However, an increase in material plasticity in the T6 state was not observed. A TEM analysis from the viewpoint of the dislocation density and the presence of the precipitates was conducted and its correlation with the mechanical results was indicated. The differences in the mechanical results between the T6 and SS states and the increase in plasticity in the latter state were discussed from the viewpoint of the electron wind and the heterogeneous microscale Joule heating theories. Material plasticity could not be increased in the case of the T6 state because the applied current DC densities were too limited to help the dislocations overcome the obstacles, especially the precipitates. However, in the case of the SS state, the applied current DC densities were not limited, and hence thanks to the heterogeneous microscale Joule heating a dislocation motion and annihilation occurred, and hence an improved material ductility. The observed lower dislocation density and more ductile morphology of the fracture surfaces confirm the above. The changes in the serrations caused by the Portevin–Le Chatelier effect were also discussed. In the case of the SS state, the current-induced hardening effect and an increase in the maximum amplitude of serrations were observed in the function of DC density; and finally, the statement that the flowing current promotes the growth and nucleation of the precipitates was proposed.

## Figures and Tables

**Table 1:** AA7075-T6 nominal chemical composition.

Element	Zn	Mg	Cu	Cr	Fe	Ti	Mn	Al
%	6.15	2.79	1.72	0.19	0.08	0.04	0.02	residual

**Table 2:** AA7075-T6 mechanical properties in the as-delivered conditions.

E (GPa)	Y_0.2_ (MPa)	UTS (MPa)	A (%)
71.6 (±6)	538 (±3)	591.2 (±1.6)	13.6 (±0.3)

**Table 3:** Experimental plan of the tensile tests.

Material State	T6	SS
DC density (A/mm^2^)	0, 5, 10, 20, 30	0, 5, 10, 20, 30
Rolling direction (deg)	0	0
Strain rate (s^−1^)	0.1	0.1
Repeatability	3	3

**Table 4:** Experimental plan of the tensile tests.

DC Density (A/mm^2^)	0	5	10	20	30
Maximum Temperature in the T6 (°C)	20	48	54	95	115
Maximum Temperature in the SS (°C)	20	36	42	70	106
Strain Rate (s^−1^)	0.1	0.1	0.1	0.1	0.1
Repeatability	3	3	3	3	3

## References

[1] Polak S., Kaczyński P., Gronostajski Z., Jaskiewicz K., Krawczyk J., Skwarski M., Zwierzchowski M., Chorzȩpa W. (2017). Warm forming of 7075 aluminum alloys. Procedia Eng..

[2] Xiao W., Wang B. (2020). Behaviors and modeling of thermal forming limits of AA7075 aluminum sheet. Arch. Civ. Mech. Eng..

[3] Liu Y., Zhu Z., Wang Z., Zhu B., Wang Y., Zhang Y. (2017). Formability and lubrication of a B-pillar in hot stamping with 6061 and 7075 aluminum alloy sheets. Procedia Eng..

[4] Gronostajski Z., Polak S., Jaśkiewicz K., Kaczyńskia P., Skwarski M., Krawczyk J., Chorzępa W., Śliz K., Uzar S. (2019). Properties of B-pillar made of aluminum 7075 in warm forming process. Procedia Manuf..

[5] Conrad H. (1998). Some effects of an electric field on the plastic deformation of metals and ceramics. Mater. Res. Innov..

[6] Roh J.H., Seo J.J., Hong S.T., Kim M.J., Han H.N., Roth J.T. (2014). The mechanical behavior of 5052-H32 aluminum alloys under a pulsed electric current. Int. J. Plast..

[7] Kim M., Song J., Huh H. (2017). Effect of Pre-strain on Tensile Properties of Al5052-H32 under an Electropulsing Condition. Procedia Eng..

[8] Salandro W.A., Jones J.J., McNeal T.A., Roth J.T., Hong S.T., Smith M.T. (2010). Formability of Al 5xxx sheet metals using pulsed current for various heat treatments. J. Manuf. Sci. Eng. Trans. ASME.

[9] Zhao K., Fan R., Wang L. (2016). The Effect of Electric Current and Strain Rate on Serrated Flow of Sheet Aluminum Alloy 5754. J. Mater. Eng. Perform..

[10] Kim M.J., Lee K., Oh K.H., Choi I.S., Yu H.H., Hong S.T., Han H.N. (2014). Electric current-induced annealing during uniaxial tension of aluminum alloy. Scr. Mater..

[11] Ross C.D., Irvin D.B., Roth J.T. (2007). Manufacturing aspects relating to the effects of direct current on the tensile properties of metals. J. Eng. Mater. Technol. Trans. ASME.

[12] Breda M., Calliari I., Bruschi S., Forzan M., Ghiotti A., Michieletto F., Spezzapria M., Gennari C. (2017). Influence of stacking fault energy in electrically assisted uniaxial tension of FCC metals. Mater. Sci. Technol..

[13] Ghiotti A., Bruschi S., Simonetto E., Gennari C., Calliari I., Bariani P. (2018). Electroplastic effect on AA1050 aluminum alloy formability. CIRP Ann..

[14] Ruszkiewicz B.J., Mears L., Roth J.T. (2018). Investigation of Heterogeneous Joule Heating as the Explanation for the Transient Electroplastic Stress Drop in Pulsed Tension of 7075-T6 Aluminum. J. Manuf. Sci. Eng. Trans. ASME.

[15] Ruszkiewicz B.J., Mears L. Investigation of the electroplastic effect through nominally equal energy deformation. Proceedings of the ASME 2018 13th International Manufacturing Science Engineering Conference MSEC.

[16] Hong S.T., Jeong Y.H., Chowdhury M.N., Chun D.M., Kim M.J., Han H.N. (2015). Feasibility of electrically assisted progressive forging of aluminum 6061-T6 alloy. CIRP Ann. Manuf. Technol..

[17] Zimniak Z., Radkiewicz G. (2008). The electroplastic effect in the cold-drawing of copper wires for the automotive industry. Arch. Civ. Mech. Eng..

[18] Bao W., Chu X., Lin S., Gao J. (2017). Electro-plastic effect on tensile deformation behavior and microstructural mechanism of AZ31B alloy. Mater. Sci. Technol..

[19] Zhao K., Fan R. (2016). The effect of pulse electric current on the mechanical properties and fracture behaviors of aluminum alloy AA5754. J. Eng. Mater. Technol. Trans. ASME.

[20] Yilmaz A. (2011). The Portevin–Le Chatelier effect: A review of experimental findings. Sci. Technol. Adv. Mater..

[21] Chen J.-Z., Zhen L., Fan L.-W., Yang S.-J., Dai S.-L., Shao W.-Z. (2009). Portevin–Le Chatelier effect in Al-Zn-Mg-Cu-Zr aluminum alloy. Trans. Nonferrous Met. Soc. China.

[22] Shibkov A.A., Denisov A.A., Zheltov M.A., Zolotov A.E., Gasanov M.F. (2014). The electric current-induced suppression of the Portevin–Le Chatelier effect in Al-Mg alloys. Mater. Sci. Eng. A.

[23] Hariharan K., Lee M.G., Kim M.J., Han H.N., Kim D., Choi S. (2015). Decoupling Thermal and Electrical Effect in an Electrically Assisted Uniaxial Tensile Test Using Finite Element Analysis. Metall. Mater. Trans. A Phys. Metall. Mater. Sci..

[24] Xu H., Liu X., Zhang D., Zhang X. (2019). Minimizing serrated flow in Al-Mg alloys by electroplasticity. J. Mater. Sci. Technol..

[25] Xu X., Zhao Y., Ma B., Zhang M. (2014). Rapid precipitation of T-phase in the 2024 aluminum alloy via cyclic electropulsing treatment. J. Alloys Compd..

[26] Liu Y., Huang M., Ma Z., Zhan L. (2016). Influence of the low-density pulse current on the ageing behavior of AA2219 aluminum alloy. J. Alloys Compd..

[27] Thevenet D., Mliha-Touati M., Zeghloul A. (2000). Characteristics of the propagating deformation bands associated with the Portevin–Le Chatelier effect in an Al-Zn-Mg-Cu alloy. Mater. Sci. Eng. A.

[28] Thevenet D., Mliha-Touati M., Zeghloul A. (1999). The effect of precipitation on the Portevin–Le Chatelier effect in an Al-Zn-Mg-Cu alloy. Mater. Sci. Eng. A.

